# Enterolithiasis in a Patient With Prior Bowel Resection

**DOI:** 10.7759/cureus.67894

**Published:** 2024-08-27

**Authors:** Michael P Willis, Matthew C Dorn

**Affiliations:** 1 Surgery, Edward Via College of Osteopathic Medicine, Blacksburg, USA; 2 General Surgery, Johnston Memorial Hospital, Abingdon, USA

**Keywords:** blind loop syndrome, intestinal pseudo-obstruction, explorative laparotomy, small bowel resection, enterolithiasis

## Abstract

Enterolithiasis is the development of intestinal stones, thought to be related to conditions that predispose to stasis and stricture of the intestines and disruption of chemical factors such as pH. It has been described in the setting of inflammatory bowel disease, intestinal tuberculosis, and prior surgery of the bowel. Our patient was a 68-year-old Caucasian female with prior bowel resection secondary to hernia repair who presented many years later with obstructive symptoms including abdominal pain, nausea and vomiting, and ultimately inability to tolerate oral intake. Initial CT scans showed nonspecific inflammation and dilation of a segment of the small bowel, unable to rule out infectious or neoplastic process, and retained fecal material. The patient was initially managed conservatively with antiemetics, antibiotics, and bowel rest. After worsening of symptoms, the patient was readmitted, and exploratory laparotomy was performed during which a mesenteric mass was discovered adjacent to an area of conglomerated bowel which contained intraluminal rock-like material. After partial bowel resection and side-by-side anastomosis, the patient showed complete clinical recovery. Emphasis is placed on the importance of considering this uncommon etiology in the differential diagnosis of obstructive symptoms. Delayed diagnosis may lead to untoward complications such as perforation, and further understanding of the pathology may lead to increased detection and earlier intervention with surgical or endoscopic management.

## Introduction

Enterolithiasis is an uncommon medical condition in which contents of chyme precipitate from solution and form rock-like structures in the bowel [1]. It is unusual in humans and typically presents in the setting of stasis syndromes such as may occur in anatomic or physiologic disturbances [1]. Enterolithiasis is classified into primary enterolithiasis, arising from within the bowel, or secondary enterolithiasis arising from outside the bowel [2]. Primary enterolithiasis is often the result of anatomic disturbance leading to areas of impaired motility or stasis of chyme, including duodenal diverticula or blind-ends from prior surgical anastomosis [1,2]. Secondary enteroliths are uncommonly described in the literature forming secondary to gallstones or, more rarely, renal calculi entering the bowel from fistulae [1].

In up to one-third of cases the stones may be visible on plain abdominal radiographs, depending on the calcium content, and more radiolucent stones may be visualized on computed tomography (CT) [2]. More radiolucent choleic acid stones are more common proximally and more radiopaque calcium salt stones tend to be found more distally [1]. It is unclear how prevalent enteroliths are in the population, as they may be passed silently unless creating a mechanical obstruction or causing a characteristic fluctuant, “tumbling” abdominal pain [1,2]. Enteroliths can cause pressure injury to the mucosa, eroding and causing hemorrhage, perforation, and diverticulitis [1,3]. In the absence of definitive radiographic evidence, diagnosis can be difficult and begins with excluding other common surgical pathologies such as obstruction secondary to hernia or adhesion, tumor, infection or inflammatory processes [4]. This case presents a 68-year-old female with prior bowel resection who presented to the emergency department (ED) with pseudo-obstructive symptoms secondary to enterolithiasis.

## Case presentation

A 68-year-old white female presented to the ED with abdominal pain, non-bloody, nonbilious emesis, and watery diarrhea for eight days. The pain was localized to the epigastrium and was non-radiating, constant, and worse with meals. The patient’s past medical history was remarkable for atrial fibrillation, cardiomyopathy, hyperlipidemia, hypertension, and alcohol use averaging five beers a day. Surgical history includes appendectomy and partial bowel resection in 2005 for obstructing hernia and a laparoscopic tubal ligation in the 1980s. On physical exam, the patient’s blood pressure was 171/82 mmHg, temperature 98°F, pulse 54 beats per minute. The abdomen was soft, and mildly distended, with hyperactive bowel sounds and tenderness to palpation in the epigastric area. There was no guarding or rebound tenderness. The patient had tried bismuth subsalicylate (Pepto-Bismol, Procter & Gamble, Cincinnati, OH, USA) at home for a few days without relief. She had transient black stools. The fecal occult blood test in the ED was negative. 

**Figure 1:**
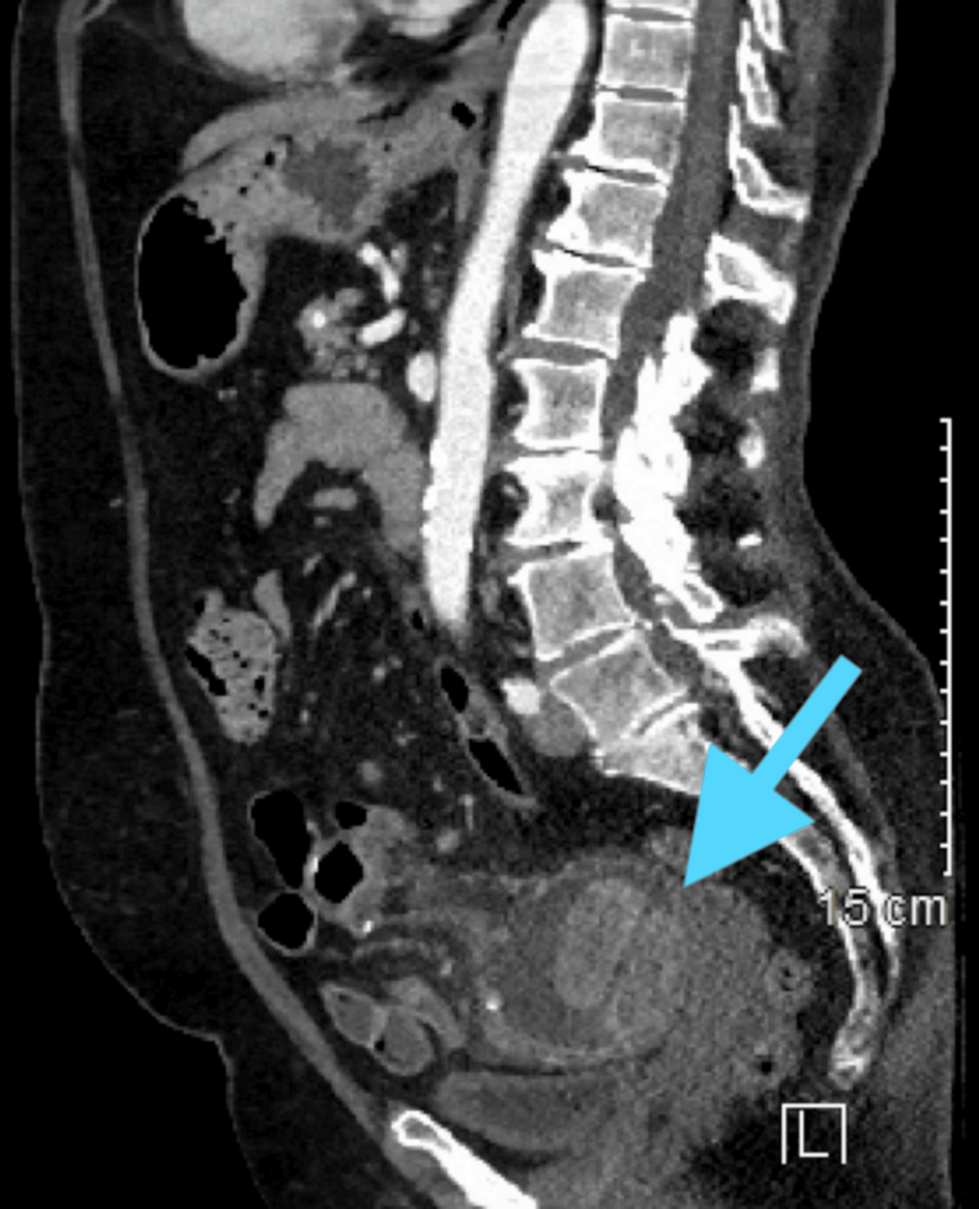
CT scan with IV contrast demonstrating retained material consistent with the enteroliths found intraoperatively.

A CT scan of the abdomen showed marked abnormal segments of the small bowel in the left hemipelvis at the site of prior postsurgical change with marked wall irregularity, mucosal enhancement, intraluminal high attenuation, and surrounding inflammatory changes; reflecting ischemic, infectious, or inflammatory colitis with underlying neoplastic process not excluded. Of note, the bowel findings included an incidental duodenal diverticulum, multiple tethered bowel loops with irregular wall thickening, and mucosal hyperenhancement with areas of intraluminal high attenuation, a dilated segment of bowel containing indeterminant 4.9 centimeters (cm) intraluminal mass-like structures and rounded inflammatory changes. Distally the small bowel was decompressed with moderate colonic stool. Abnormal mesenteric lymph nodes were noted. A representative image from this scan is included in Figure 1.

Laboratory studies were unremarkable except for mildly elevated alkaline phosphatase at 143 units per liter and positive leukocyte esterase on urinalysis. The patient was admitted due to abdominal pain and for surgical evaluation.

**Figure 2:**
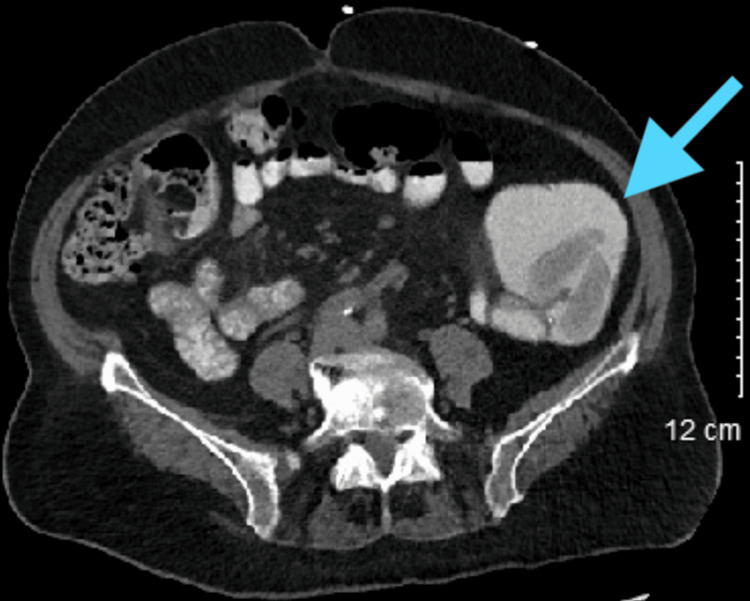
An abdominal CT scan with oral contrast demonstrating the enteroliths found intraoperatively.

The differential diagnoses for this patient included colitis, partial bowel obstruction, and possible neoplastic process. She was started on ceftriaxone (Rocephin, F. Hoffmann-La Roche Ltd., Basel, Switzerland) and metronidazole (Flagyl, Pfizer Inc., New York City, NY) for coverage of possible urinary tract infection and colitis, ondansetron (Zofran, Pfizer Inc.) for nausea, and general surgery was consulted. A CT scan with oral contrast was performed which showed a patulous small bowel, nonspecific inflammation of the small bowel with irregular mural thickening and mesenteric stranding, retained solid food material, but did not show evidence of obstruction. A representative image of this scan is shown in Figure 2. The patient was tolerating oral intake and was discharged on ciprofloxacin (Cipro, Bayer HealthCare Pharmaceuticals Inc., North Rhine-Westphalia, Germany) and metronidazole with outpatient follow-up.

The patient returned to the ED two days later with persistent nausea and vomiting, chills, blood-tinged emesis, abdominal pain, hypertensive urgency, unable to tolerate food, and two days without bowel movement. On repeat admission, her blood pressure was 226/112 mmHg with a pulse of 58 bpm and a temperature of 98°F. The abdominal pain was described as intermittently sharp and crampy, localized to the epigastrium and middle abdomen. There was epigastric tenderness to light palpation and left lower quadrant pain to deep palpation. She was offered a nasogastric tube decompression but declined. Due to the inability to tolerate oral intake, refractory nausea, and nonspecific findings on imaging the decision was made with the patient to pursue exploratory laparotomy.

Extensive intra-abdominal adhesions were encountered and lysed. In the mid portion of the small bowel, there was a conglomeration of small bowel adhered together with an internal hernia and stricture. A firm lymph node or mass was palpated within the mesentery of this region. There was evidence of intraluminal obstructing masses, which were removed by proximal enterotomy and discovered to be a pair of enteroliths approximately 5 cm in width. Approximately 2 feet of small bowel was removed including the mesenteric mass and reapproximated with side-by-side anastomosis. The patient tolerated the procedure well. Post-operative recovery was unremarkable, and the patient was discharged on postoperative day 5. At the outpatient follow-up two weeks later, the patient endorsed the complete resolution of symptoms and continued to be symptom-free at a two-month outpatient follow-up.

**Figure 3:**
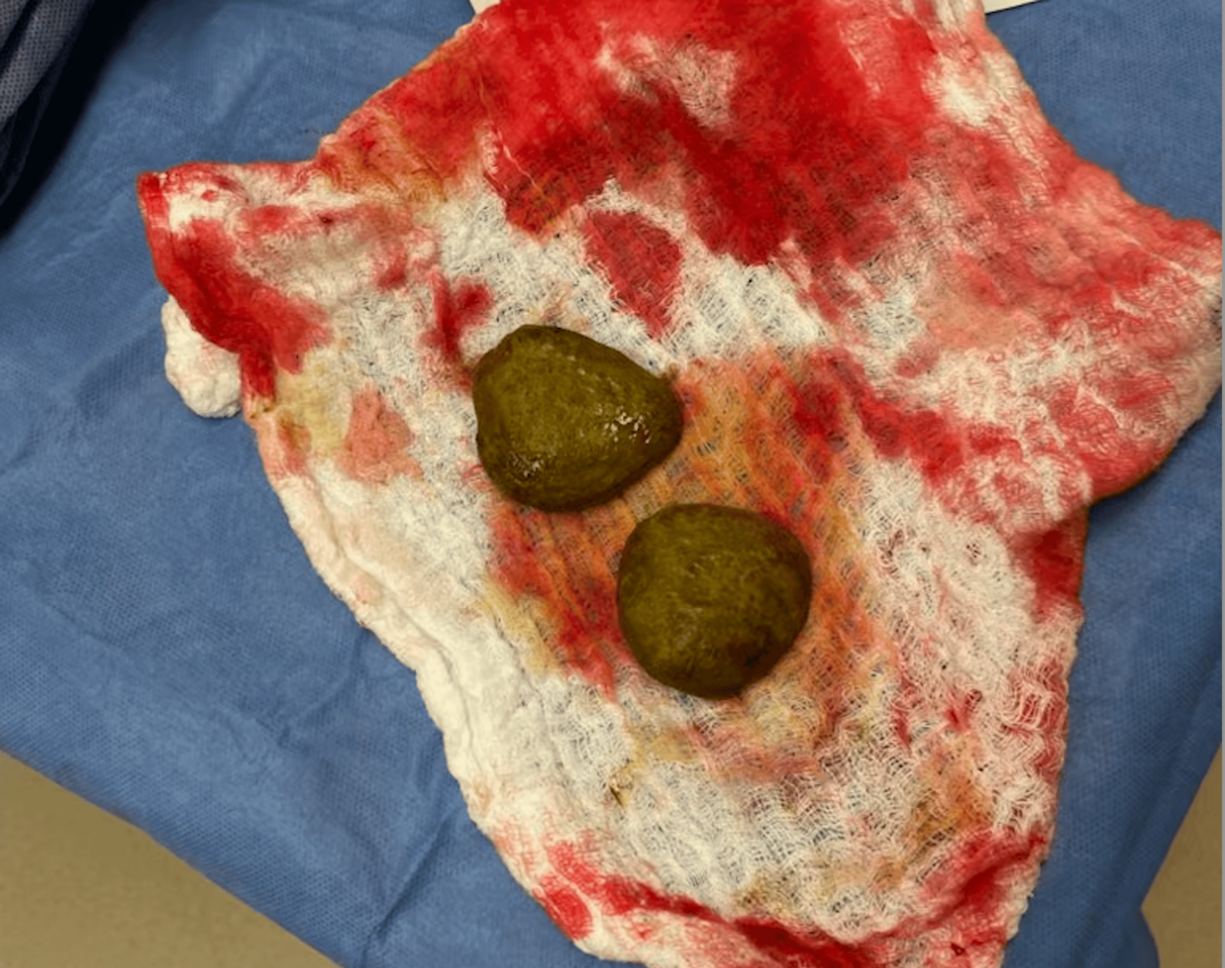
Intraoperative photograph of the enteroliths extracted from the patient's small bowel.

The enteroliths and resected small bowel were sent to pathology. The small bowel showed previous side-by-side anastomosis with a blind-end which adhered to the adjacent bowel, forming a loop with a transmural defect and an area of grossly cobblestone-like mucosal erosion with underlying dense chronic inflammation, likely due to the enteroliths, negative for active enteritis or ischemia or neoplasm. The enteroliths were composed of hard, brown-green, crystalline material and measured 5 x 5 x 2.3 cm and 5.4 x 5 x 2.4 cm. A photo taken of the stones during the operation is included in Figure 3.

## Discussion

Enterolithiasis is an uncommon condition that has been described as associated with intestinal stasis syndromes and anatomic abnormalities [1]. While it has been described in association with prior enteroanastamosis [2] and blind-end pouches [5], cases remain uncommonly reported and frequently there is a well-defined opacity on radiographic examination. While the premise of association with stasis is generally accepted, other risk factors such as alterations in luminal pH, gut microbiota, or mineral content of the diet may play a role [1]. The classification of enteroliths is summarized in Table 1. As described previously, “primary” and “secondary” relate to the site of origin of the conglomeration, where “true primary” is within the bowel, “false primary” is by means of ingestion, and “secondary” is from an endogenous source outside of the bowel [1,2].

**Table 1:** Classification of enteroliths. Information from references [1,2]. IBD = inflammatory bowel disease.

Type	Composition	Etiology
True Primary	Choleic acid, calcium stones	Stasis, stricture (IBD), postoperative blind loops, pH imbalance, bacterial dysbiosis
False Primary	Bezoars, Varnish stones, insoluble materials	Trichotillomania, Varnish drinking, insoluble salts
Secondary	Gallstones, Kidney stones	Gallstone ileus, cholecystoduodenal fistula, duodenal renal stone erosion

While it is thought that side-to-side or end-to-side anastomosis can lead to enteroliths secondary to the formation of blind-loops, cases are described with prior end-to-end anastomosis and enterotomy [5,6]. Select patient factors may contribute to the degradation of bowel function at the site of anastomosis, leading to the formation of defects in the integrity of the wall [5]. Strictures from inflammatory bowel diseases in developed countries, or intestinal tuberculosis in developing countries have lent further support to the stasis hypothesis [7]. It has been described in neonatal atresia syndromes, though it seems that the presence of anatomic blind-ends is insufficient to predispose to the development of enteroliths, and a change in pH due to retention of alkaline urine may contribute [8].

Our understanding of the process appears to be incomplete, as enteroliths are described in the absence of known predisposing disease or surgery [9]. Enteroliths are described as more common in equids, and risk factors have been identified including consumption of alfalfa, which is lower in fiber and contains more minerals, and is associated with increases in colonic pH [10]. However, a retrospective analysis failed to demonstrate the efficacy of interventions aimed at preventing their recurrence, suggesting that there are patient factors in equids that remain to be clarified [11].

Our case depicts the diagnostic dilemma that can be encountered by clinicians when faced with nonspecific obstructive symptoms and unclear radiographic data. Although one can retrospectively appreciate the enteroliths on our case’s CT as above, interpretation at the time of admission given the vague presentation and data demonstrates the diagnostic challenges including ruling out more common surgical pathology. While a history of prior anastomosis may serve to increase the clinician’s index of suspicion for the development of enteroliths, there are several factors in this case that limit the ability to interpret such an association as etiologic: the first CT scan noted the presence of a duodenal diverticulum, which is also described as a risk factor for developing enteroliths [1], and there may be some relationship between ethanol consumption and altered motility [12] or intestinal dysbiosis [13]. In our case, chemical analysis is not routinely performed and likely would not have contributed to the immediate management, though our knowledge of the pathogenesis may be improved with its use in future cases.

There is a paucity of data regarding the management of enteroliths, and a query of “enterolithiasis” on UpToDate returns no results. Changing the query to “fecalith” returns articles on appendicitis. As the detection and reporting of enteroliths increases with time and improvement in the availability of imaging and increased clinical suspicion we may better understand its pathogenesis and management [1]. The use of intraoperative crushing and manual milking of stones is described as a less invasive strategy if possible [9] and the use of endoscopic lithotripsy of enteroliths has been reported [5]. Further reporting and investigation may help clinicians in the diagnosis and management of enterolithiasis.

## Conclusions

Enterolithiasis poses a diagnostic dilemma as it is uncommon enough to be low on the index of clinical suspicion, particularly when imaging is diagnostically inconclusive. Our case demonstrates the value of the clinical presentation, differential diagnosis, and clinical decision-making involved. A thorough understanding of patient factors, further understanding of the pathology of enteroliths, and further trials of surgical and endoscopic interventional modalities are essential to developing effective and patient-centered management.
